# Comprehensive genomic transcriptomic tumor-normal gene panel analysis for enhanced precision in patients with lung cancer

**DOI:** 10.18632/oncotarget.24973

**Published:** 2018-04-10

**Authors:** Shahrooz Rabizadeh, Chad Garner, John Zachary Sanborn, Stephen C. Benz, Sandeep Reddy, Patrick Soon-Shiong

**Affiliations:** ^1^ NantOmics, LLC, Culver City, CA, USA; ^2^ NantHealth, Inc., Culver City, CA, USA

**Keywords:** precision medicine, tumor-normal sequencing, lung cancer, somatic variants, tumor sequencing

## Abstract

A CMS approved test for lung cancer is based on tumor-only analysis of a targeted 35 gene panel, specifically excluding the use of the patient’s normal germline tissue. However, this tumor-only approach increases the risk of mistakenly identifying germline single nucleotide polymorphisms (SNPs) as somatically-derived cancer driver mutations (false positives). 621 patients with 30 different cancer types, including lung cancer, were studied to compare the precision of tumor somatic variant calling in 35 genes using tumor-only DNA sequencing versus tumor-normal DNA plus RNA sequencing. When sequencing of lung cancer was performed using tumor genomes alone without normal germline controls, 94% of variants identified were SNPs and thus false positives. Filtering for common SNPs still resulted in as high as 48% false positive variant calling. With tumor-only sequencing, 29% of lung cancer patients had a false positive variant call in at least one of twelve genes with directly targetable drugs. RNA analysis showed 18% of true somatic variants were not expressed. Thus, sequencing and analysis of both normal germline and tumor genomes is necessary for accurate identification of molecular targets. Treatment decisions based on tumor-only analysis may result in the administration of ineffective therapies while also increasing the risk of negative drug-related side effects.

## INTRODUCTION

In 2016, the Centers for Medicare and Medicaid Services (CMS) authorized coverage of a tumor-only DNA sequencing-based test of 35 genes intended to inform lung cancer treatment. The test (MolDX: L36194) is defined as a *“single test using tumor tissue only (i.e., not matched tumor and normal) that does not distinguish between somatic and germline alterations”*. However, this tumor-only approach has been reported by others to increase the risk of mistakenly identifying germline mutations as somatically-derived genetic changes and potential cancer driver mutations (“false positives”) [1, 2]. Jones *et al.* [1] recently showed that tumor-only sequencing and analysis can result in a significant number of inherited germline variants being identified as somatic variants. The pitfalls of tumor-only sequencing were further demonstrated by Teer *et al.* [2] in their recent investigation of a range of filtering methods for tumor-only sequencing. Bioinformatics and statistical methods have been developed specifically for the purpose of identifying somatic mutations with tumor-only sequencing data, including an extension to a well-known variant calling algorithm that incorporates new statistical and machine learning components [3], as well as a method that leverages ancestry information and allele fraction to improve the identification of somatic and germline variants [4]. While these bioinformatics and statistical methods have reduced false positive rates, the rates remain unacceptably high for use with tumor-only sequencing in a clinical setting. Others have shown that false positive rates associated with tumor-only sequencing can be further reduced by molecular pathologist review of all putative somatic variants [1, 5]. The problem of identifying mutations of germline origin from tumor-only sequencing has recently been highlighted [6] and tumor-normal sequencing and analysis was shown to be significantly better at identifying inherited cancer susceptibility mutations than guideline-based germline testing [7].

Based on these concerns of false positives of tumor-only gene panel analysis, we sought to demonstrate (i) the enhanced precision afforded by simultaneously analyzing both tumor and germline sequences, and thereby improving the confidence with which clinically druggable mutations can be identified, (ii) bioinformatic filtration of polymorphisms from tumor-only sequence analysis does not match the precision of tumor-normal genomic analysis, (iii.) mRNA expression of any true somatic mutation provides the critical second line of support for the mutation’s druggability. In the present study, DNA sequencing of tumor and normal germline genomes of the 35-gene panel authorized for coverage by CMS from 45 lung cancer patients and 621 total cancer patients with 33 cancer types was used to quantify the rate of false positive tumor somatic variants originating from the use of the tumor-only sequencing approach. Potential increase in precision stemming from expression analysis of alterations in these 35 genes by RNA sequencing was also assessed.

## RESULTS

### Identification of tumor somatic single nucleotide variants (SNVs)

Whole-genome DNA sequencing of 45 lung cancer patients’ tumor and normal (germline) genomes resulted in the identification of 802 missense or nonsense protein-altering SNVs in the panel of 35 genes associated with lung cancer etiology. The panel included 25 genes considered somatic tumor drivers (tumor driver gene panel), and 10 genes known to affect inherited cancer risk (inherited risk gene panel; Table 1). Among the 45 lung cancer patients, the total of 746 SNVs occurred at 147 unique SNV sites. All 746 variants were present in the tumor genomes. Bioinformatic analysis of tumor and normal germline DNA sequence showed that 701 of the 746 SNVs (94%) originated in the germline, and the remaining 45 SNVs (6%) originated in somatic tissue. Applying the same gene panel to the analysis of 621 cancer patients’ with 33 cancer types, tumor-normal sequencing analysis resulted in the identification of 10,704 missense or nonsense protein-altering SNVs. There were 919 unique SNVs sites that contributed to the 10,704 SNVs identified. Analysis of each patient’s tumor and normal germline genome determined that 10,149 (95%) of the SNVs were of germline origin, while the remaining 555 (5%) SNVs were of somatic origin.

**Table 1:** Identification of tumor somatic single nucleotide variants (SNVs)

	Numbers of variants in patients with all cancer types			Numbers of variants in lung cancer patients only		
Gene	Unique	Germline	Somatic	Unique	Germline	Somatic
**Tumor driver gene panel**						
ALK	32	1317 (99%)	14 (1%)	6	93 (99%)	1 (1%)
BRAF	23	5 (15%)	29 (85%)	3	0 (0%)	3 (100%)
CDKN2A	22	35 (71%)	14 (29%)	5	2 (40%)	3 (60%)
CEBPA	8	2 (25%)	6 (75%)	0	0	0
DNMT3A	22	12 (52%)	11 (48%)	1	1 (100%)	0 (0%)
EGFR	29	315 (95%)	16 (5%)	6	15 (71%)	6 (29%)
ERBB2	38	921 (98%)	15 (2%)	7	68 (100%)	0 (0%)
EZH2	12	117 (94%)	8 (6%)	1	3 (100%)	0 (0%)
FLT3	25	846 (99%)	5 (1%)	6	64 (98%)	1 (2%)
IDH1	9	85 (94%)	5 (6%)	2	2 (100%)	0 (0%)
IDH2	10	9 (64%)	5 (36%)	0	0	0
JAK2	18	37 (88%)	5 (12%)	0	0	0
KIT	19	138 (93%)	10 (7%)	5	8 (62%)	5 (38%)
KMT2A	57	72 (80%)	18 (20%)	3	2 (67%)	1 (33%)
KRAS	16	3 (4%)	77 (96%)	4	0 (0%)	7 (100%)
MET	28	58 (84%)	11 (16%)	5	7 (87%)	1 (13%)
NOTCH1	59	143 (89%)	17 (11%)	8	6 (75%)	2 (25%)
NPM1	2	1 (50%)	1 (50%)	0	0	0
NRAS	10	1 (5%)	18 (95%)	0	0	0
PDGFRA	24	169 (92%)	14 (8%)	2	9 (100%)	0 (0%)
PDGFRB	28	98 (92%)	8 (8%)	8	11 (92%)	1 (8%)
PGR	31	377 (96%)	15 (4%)	7	21 (91%)	2 (9%)
PIK3CA	31	96 (54%)	82 (46%)	2	6 (86%)	1 (14%)
PTEN	33	780 (97%)	24 (3%)	2	56 (100%)	0 (0%)
RET	22	244 (96%)	9 (4%)	7	21 (100%)	0 (0%)
**Total**	**608**	**5881**	**437**	**90**	**395**	**34**
**Inherited risk gene panel**						
APC	85	692 (92%)	58 (8%)	7	48 (98%)	1 (2%)
BMPR1A	5	334 (99%)	2 (1%)	1	17 (100%)	0 (0%)
EPCAM	13	464 (100%)	0 (0%)	3	37 (100%)	0 (0%)
MLH1	15	295 (99%)	4 (1%)	4	26 (96%)	1 (4%)
MSH2	23	40 (89%)	5 (11%)	4	5 (100%)	0 (0%)
MSH6	25	273 (98%)	7 (2%)	2	18 (100%)	0 (0%)
PMS2	44	1558 (99%)	10 (1%)	13	110 (97%)	3 (3%)
POLD1	30	208 (97%)	7 (3%)	4	11 (100%)	0 (0%)
POLE	58	398 (96%)	18 (4%)	16	34 (92%)	3 (8%)
STK11	13	6 (46%)	7 (54%)	3	0 (0%)	3 (100%)
**Total**	**311**	**4268**	**118**	**57**	**306**	**11**

For lung cancer patients, just 8% and 3% of SNVs were of somatic origin in the tumor driver gene panel and inherited risk gene panels, respectively. Among all cancer patients, the percentage of SNVs representing somatic changes was 7% and 3% for genes in the tumor driver gene panel and inherited risk gene panel, respectively. A greater percentage of somatic variants was expected to be observed among the 25 genes that are known to harbor somatic cancer driver mutations. There was significant variation in the number of SNVs observed in each gene. The number of unique SNV sites was strongly correlated with the size of the gene protein-coding sequence (*p* value < 10^–9^, *R*^2^ = 0.70 for all cancer types). However, there was no correlation between the number of germline, somatic, or total variants and the size of the gene (all *p*-values > 0.40). The degree of association between each gene and the cancer outcomes is a likely determinant of the variation in SNV counts observed between genes as well as the natural population genetic variation present in each gene. Furthermore, specific cancer driver SNVs are enriched among the patients. The copy number state of each gene was not considered when evaluating the number and distributions of somatic and germline variants within and between genes.

The small number of unique variants compared to total variants illustrates the presence of common SNVs that are observed in many genomes in the study population of cancer patients. There were 21 variants that had allele frequencies > 0.02 in the sample of 621 cancer patients, 17 of which were common germline SNPs and 4 of which were common somatic driver mutations (2 in KRAS and 2 in PIK3CA). All 21 common variants are archived in the single nucleotide polymorphism database (dbSNP) of genetic polymorphisms. Among all patients, 645 of the 919 total unique variants (70%) were observed only once. Three SNVs were of both germline and somatic origin.

**Figure 1:**
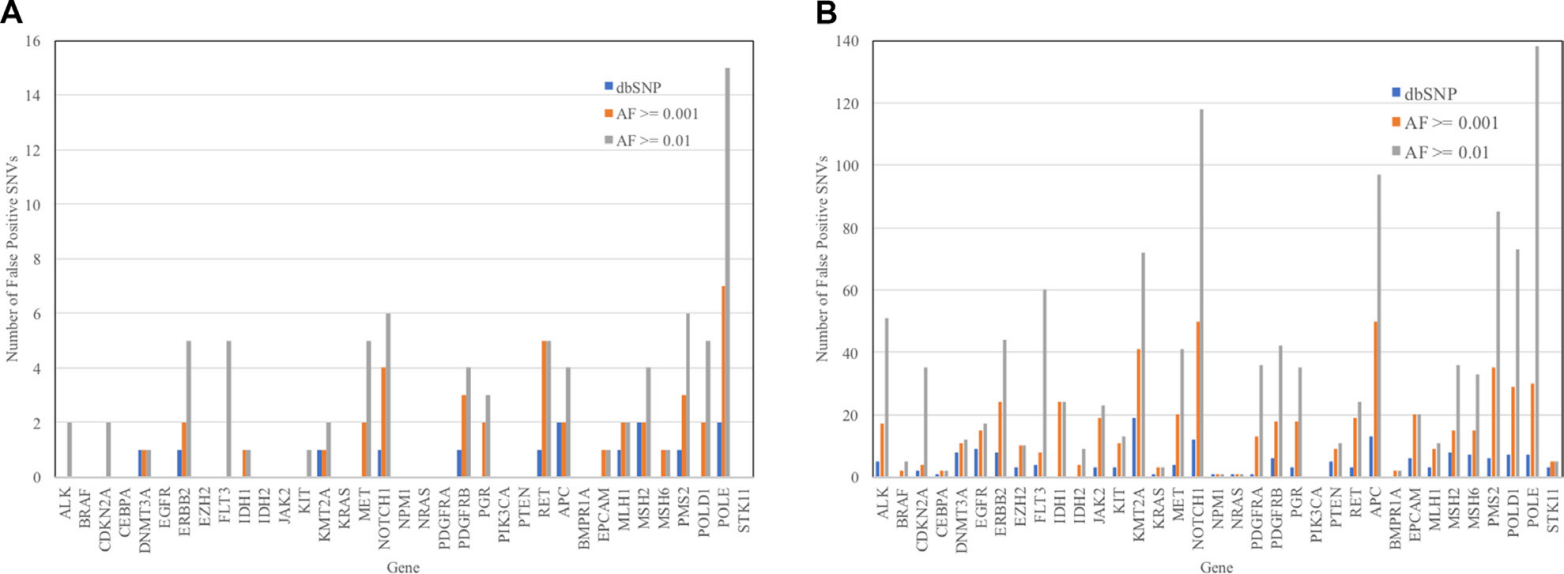
Number of false positive missense or nonsense protein-altering SNVs from tumor sequence analysis of 35 genes in 45 lung cancer patients (**A**) and 621 total cancer patients (**B**) after filtering of SNVs based on three criteria. False-positive results are defined as SNVs found in tumors that are of inherited germline origin. Filters used included the following exclusion criteria: 1) SNPs present in the dbSNP database (dbSNP); 2) SNPs with reported population allele frequency ≥ 0.01 (AF ≥ 0.01); and 3) SNPs with a reported population allele frequency ≥ 0.001 (AF ≥ 0.001).

Tumor genome sequencing alone (without comparison to the normal germline genome) of the 45 lung cancer patients would identify 746 missense and nonsense protein-altering SNVs (Table 1). In the context of tumor molecular profiling, any SNV of germline origin that is categorized to be of somatic origin constitutes a false positive result. Without any filtering of putative germline variants, false positive rates of approximately 94% are expected, given the data presented in Table 1. Figure 1 shows the number of false positive results that would occur among the 45 lung cancer patients (Figure 1A) and the 621 all cancer patients (Figure 1B) for each gene with three different SNV filtering criteria: 1) removing all SNVs that are found in the dbSNP database; 2) removing all SNVs with reported population allele frequencies ≥ 0.01 (1%); and 3) removing all SNVs with reported population allele frequency ≥ 0.001 (0.1%). (An additional three SNVs that had no reported population allele frequencies but were common germline SNVs among the cancer patients and were present in dbSNP were also removed). The largest numbers of false positive results occurred using an allele frequency threshold of 0.01. The number of false positives could be reduced by half in most genes by reducing the allele frequency filtering threshold to 0.001. The precision of most publicly-available population allele frequency estimates did not exceed 0.0001 so further reductions in the population allele frequency threshold had a nominal effect on the number of false positive SNVs.

Excluding all of the SNPs that were present in the dbSNP database resulted in the lowest numbers of false positive SNVs. However, the improved false positive rate came at the cost of an increased false negative rate, as many true tumor somatic SNVs were excluded. Excluding all SNVs present in dbSNP resulted in 17 false negatives among 45 true tumor somatic variants observed in the 45 lung cancer patients (38%), and 245 false negatives out of the 555 true somatic variants among the lung cancer patients (44%). Using the 0.001 allele frequency threshold filter, there were 41 false positive results (5% of the 746 total SNVs observed and 48% of the 86 SNVs remaining after filtering) and zero false negative results among lung cancer patients. The same filtering threshold resulted in 554 false positive results (5% of the 10,704 total SNVs observed and 50% of the 1,107 SNVs remaining after filtering) and zero false negative results among all 621 cancer patients.

### Consequences of the tumor-only sequencing approach

**Figure 2:**
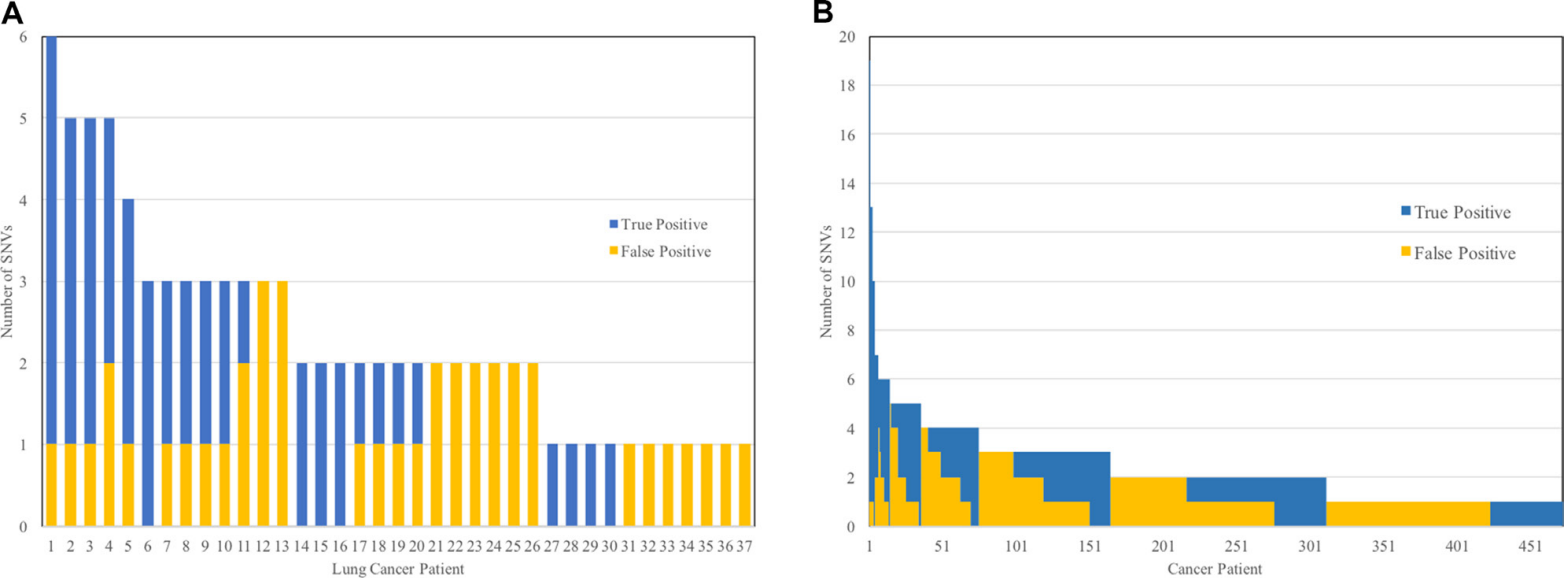
Number of true positive and false positive results among lung cancer patients (**A**) and all cancer patients (**B**) after excluding SNVs with a population allele frequency of 0.001 or greater. There were 8 of the 45 lung cancer patients and 149 of the 621 total patients not shown in the histograms that had zero variants in the 35 genes after filtering based on allele frequency.

After filtering to remove all SNVs with a population allele frequency ≥ 0.001, 37 of the 45 lung cancer patients, and 472 of the 621 all cancer patients had at least one missense or nonsense protein-altering SNV in the panel of 35 genes. The 7 lung cancer and 149 total patients without SNVs after filtering did not have any true somatic variants, showing that the population allele frequency filter did not produce false negative results. Figure 2 shows the number of true positive (i.e., the number of tumor somatic SNVs) and false positive SNVs (i.e., the number of inherited germline SNVs) for lung cancer (Figure 2A) and all patients (Figure 2B) that had at least one SNV remaining after filtering. The average numbers of SNVs were 1.91 and 1.84, for lung cancer and all cancer patients, respectively. One patient with 39 somatic SNVs was excluded from Figure 2B for presentation purposes. In lung cancer patients, 29 of the 45 patients (65%) had at least one false positive SNV, and 15 patients had only false positive SNVs (33%), without any true positive results. While only 5% of the total SNVs found among lung cancer patients were false positives after filtering at a population allele frequency of 0.001 (41 false positives out of 802 total SNVs discovered), the SNVs were distributed across 65% of the patients. The majority of the 802 SNVs discovered are common variants that are excluded by filtering. These results highlight the impact of rare germline mutations on the rate of false positive discoveries. In the full study population, 365 of the 621 patients (59%) had at least one false positive SNV, yielding an average of 0.91 false positives per patient. Only false positive SNVs, without true positive results, were present in 193 of 621 patients (31%).

False positive SNVs can have a direct detrimental impact on patient care. Table 2 shows 12 druggable genes, the specific drugs that target each of the genes when they are somatically mutated, and the number of patients with at least 1 false positive SNV observed in each of the genes. Furthermore, the cost and possible adverse health effects associated with each drug are shown to illustrate the financial and clinical implications of prescribing a drug based on a false positive result. Although these are non-classic variants, the patient population have often failed one or more lines of therapy and their physicians are more likely to choose to target these alterations than ignore them in this clinical setting.

**Table 2:** False positive results in drug targetable genes

Drug	Gene targeted by drug	Number of patient with at least one false positive variant after each SNV filter						Approximate Drug cost per patients^a^	Warning and precautions (FDA Label)
		No Filter		AF ≥ 0.01		AF ≥ 0.001			
		All	LC	All	LC	All	LC		
Crizotinib	ALK	621	45	50	2	16	0	$18,349.50	Pneumonitis, Hapatic Abnormalities, QT Prolongation
Alectinib								$15,976.33	Hepatotoxicity, ILD/Pneumonitis, Bradycardia, Myalgia, CPK elevation, EFT
Ceritinib								$18,964.13	GI toxicity, Hepatotoxicity, ILD/Pneumonitis, QT prolongation, Hyperglycemia, Bradycardia, Pancreatitis, EFT
Brigatinib								$15,960.00	ILD/Pneumonitis, HTN, Bradycardia, Visual disturbance, CPK elevation, Pancreatic enzyme elevation, Hyperglycemia, EFT
Vemurafenib	BRAF	5	0	5	0	2	0	$13,020.94	Hypersensitivity, Dermatologic reactions, QT Prolongation, Hepatotoxicity, Ophthalmologic reactions, Renal failure, EFT
Dabrafenib								$11,412.43	Febrile drug reaction, Hyperglycemia, Uveitis and Iritis, G6PD deficiency, EFT
Cobimetinib								$7,856.04^a^	Hemorrhage, Cardiomyopathy, Dermatologic reactions, Retinopathy and RVO, Hepatotoxicity, Rhabdomyolysis, Photosensitivity, EFT
Trametinib								$12,450.00	Cardiomyopathy, RPED, RVO, ILD, Skin toxicity, EFT
Azacitidine	DNMT3A	12	1	12	1	11	1	$2,221.81^c^	Cytopenias, Hepatotoxicity, Renal abnormalities, EFT
Decitabine								$3,967.37^c^	Cytopenias, EFT
Erlotinib	EGFR	303	15	16	0	14	0	$9,390.44	ILD, Renal failure, Hepatotoxicity, GI perforations, Bullous and skin disorders, CVA, MAHA, Ocular disorders, EFT
Afatinib								$9,060.85	Diarrhea, Bullous and skin disorders, ILD, Hapatic toxicity, Keratitis, EFT
Gefitinib								$9,117.36	Diarrhea, Bullous and skin disorders, ILD, Hapatic toxicity, Keratitis, EFT, GI perforation
Neratinib	ERBB2	544	37	43	5	24	2	$12,600.00	Diarrhea, Hepatotoxicity, EFT
Lapitinib								$6,314.31	Decreased LVEF, Hepatotoxicity, Diarrhea, ILD and pneumonitis, QT interval prolongation, EFT
Ruxolitinib	JAK2	37	0	23	0	19	0	$12,932.64	Cytopenias, Infection
Imatinib	KIT	135	8	13	1	11	0	$23,152.39	Edema, Cytopenias, CHF and LV dysfunction, Hepatotoxicity, Hemorrhage, GI perforations, Cardiogenic shock, Bullous, Hypothyroidism, EFT
Dasatinib								$16,084.02	Myelosuppression, Thrombocytopenia, Fluid retention, QT Prolongation, CHF, LV dysfunction, MI, EFT
Regorafenib								$17,857.80^d^	Hemorrhage, Dermatological toxicity, HTN, Cardiac ischemia and infarcation, RPLS, GI perforation, Wound healing complications, EFT
Crizotinib	MET	58	7	41	5	20	2	$18,349.50	Pneumonitis, Hapatic Lab Abnormalities, QT Interval Prolongation, EFT
Cabozantinib								$18,191.26	Hemorrhage, GI perforations, Thrombotic events, HTN, Diarrhea, PPES, RPLS, EFT
Axitinib	PDGFRA	160	9	36	0	13	0	$16,416.28	Hemorrhage, GI perforations, Thrombotic events, HTN, Hypothyroidism, RPLS, EFT
Regorafenib								$17,857.80^d^	Hemorrhage, Dermatological toxicity, HTN, Cardiac ischemia and infarcation, RPLS, GI perforation, Wound healing complications, EFT
Axitinib	PDGFRB	89	9	42	4	18	3	$16,416.28	Hemorrhage, GI perforations, Thrombotic events, HTN, Hypothyroidism, RPLS, EFT
Regorafenib								$17,857.80^d^	Hemorrhage, Dermatological toxicity, HTN, Cardiac ischemia and infarcation, RPLS, GI perforation, Wound healing complications, EFT
Idelalisib	PIK3CA	96	6	0	0	0	0	$5,721.26^e^	Cutaneous reactions, Anaphylaxis, Neutropenia, EFT
Everolimus								$17,013.54	Pneumonitis, Infections, Oral ulceration, EFT
Cabozantinib	RET	217	18	22	5	19	5	$18,191.26	Hemorrhage, GI perforations, Thrombotic events, HTN, Diarrhea, PRES, RPLS, EFT
Vandetinib								$15,445.43	QT prolongation, Skin reactions, ILD, Ischemic cerebrovascular events, Hemorrhage, Diarrhea, HTN, RPLS, EFT
Total number of unique patients with a FP SNV		621 (100%)	45 (100%)	303 (49%)	23 (51%)	167 (27%)	13 (29%)		

AF = population allele frequency; All = patients with all 30 cancer types; LC = lung cancer patients only; ILD = Interstitial lung disease; EFT = Embryofetal toxicity; RVO = Retinal vein occlusion; RPED = Retinal pigment epithelial dystrophy; CVA = Cerebrovascular accident; MAHA = Microangiopathic hemolytic anemia; GI = Gastrointestinal; LVEF = Left ventricular ejection fraction; MI = Myocardial infarction; RPLS = Reversible posterior leukoencephalopathy syndrome; PRES = Posterior reversible encephalopathy syndrome; HTN = Hypertension (including hypertensive crisis);

^a^Average wholesale price for 30 days unless otherwise noted.

^b^Drug not given continuously.

^c^Single cycle based on body surface area of 2.02.

^d^Based on 21 days on and 7 days off schedule.

^e^Based on 14 days on and 14 days of schedule.

### Expression of somatic single nucleotide variants

RNA sequencing data allowing assessment of the expression of the tumor somatic SNVs was available from 26 lung cancer patients and 378 of all patients. Table 3 shows the total number of somatic SNVs assessed, the number of somatic SNVs that were not expressed, and the number of patients with a somatic SNV that was not expressed. A significant percentage of SNVs were not expressed: 18% (7 out of 39 SNVs) for lung cancer patients, and 15% (75 out of 517 SNVs) for all cancer patients. There was substantial variation in the percent of expressed tumor somatic variants between genes. Nearly 80% or more of SNVs in *FLT3*, *PDGFRA*, *PGR*, and *RET* were not expressed among all cancer patients. In the study population, 9% of lung cancer patients (6 of all 26 patients with tumor RNA sequencing data) and 13% of all cancer patients (51 of 378 total cancer patients with tumor RNA sequencing data) had at least one true tumor somatic SNV that was not expressed in the messenger RNA. There were 4 tumor somatic SNVs in 4 lung cancer patients that were not expressed in the twelve genes that are targets for specific drugs shown in Table 2. There were 33 of all cancer patients with tumor somatic SNVs that were not expressed in the RNA. Treatment decisions based on DNA analysis alone might thus result in administration of ineffective therapies.

**Table 3:** Expression of somatic SNVs

	All cancer types			Lung Cancer Only		
Gene	Somatic SNVs	Somatic SNVs not expressed (%)	Patients with not expressed SNV	Somatic SNVs	Somatic SNVs not expressed (%)	Patients with not expressed SNV
ALK	13	10 (76%)	9	0	0	0
BRAF	24	0 (0%)	0	2	0 (0%)	0
CDKN2A	13	2 (15%)	2	3	0 (0%)	0
CEBPA	5	1 (20%)	1	0	0	0
DNMT3A	11	1 (9%)	1	0	0	0
EGFR	16	1 (6%)	1	6	0 (0%)	0
ERBB2	14	1 (7%)	1	0	0	0
EZH2	8	0 (0%)	0	0	0	0
FLT3	5	4 (80%)	4	1	1 (100%)	1
IDH1	5	0 (0%)	0	0	0	0
IDH2	5	0 (0%)	0	0	0	0
JAK2	5	1 (20%)	1	0	0	0
KIT	8	5 (63%)	5	4	2 (50%)	2
KMT2A	18	2 (11%)	2	1	0 (0%)	0
KRAS	70	2 (3%)	2	6	1 (17%)	1
MET	11	3 (27%)	3	1	1 (100%)	1
NOTCH1	16	1 (6%)	1	2	0 (0%)	0
NPM1	1	0 (0%)	0	0	0	0
NRAS	15	0 (0%)	0	0	0	0
PDGFRA	14	11 (79%)	8	0	0	0
PDGFRB	8	3 (38%)	3	1	1 (100%)	1
PGR	14	13 (93%)	11	1	1 (100%)	1
PIK3CA	75	0 (0%)	0	1	0 (0%)	0
PTEN	23	1 (4%)	1	0	0	0
RET	9	7 (78%)	6	0	0	0
APC	54	4 (7%)	4	1	0 (0%)	0
BMPR1A	1	0 (0%)	0	0	0	0
EPCAM	0	0	0	0	0	0
MLH1	4	0 (0%)	0	1	0 (0%)	0
MSH2	5	0 (0%)	0	0	0	0
MSH6	7	1 (14%)	1	0	0	0
PMS2	10	0 (0%)	0	3	0 (0%)	0
POLD1	7	0 (0%)	0	0	0	0
POLE	16	1 (6%)	1	2	0 (0%)	0
STK11	7	0 (0%)	0	3	0 (0%)	0
**Total**	517	75 (15%)	51 unique	39	7 (18%)	6 unique

There were 3,698 true germline variants across all cancer types with RNA sequencing coverage. The percentage of germline variants that were not expressed was 9% (337 germline variants not expressed of 3,698 total variants). Among lung cancer patients, 11% of true germline variants were not expressed (23 variants not expressed of 215 total).

## DISCUSSION

Currently, two sequencing-based approaches are available to identify a patient’s tumor somatic variation. In the first approach, the tumor DNA representing a targeted gene panel, the exome, or whole genome is sequenced, and putative germline variation is filtered based on a reference genome and the characteristics of the individual genomic variants discovered in the tumor (termed tumor-only analysis). Identification of a genomic variant in a population genetic database at an appreciable allele frequency is a common filtering criterion for determining if a variant is of inherited germline origin [2]. The second and more precise approach as shown in this study, is to use the patient’s own germline genome as the precise control (rather than a reference genome for filtration) for distinguishing the inherited germline variants from those that are somatically derived (termed tumor-normal analysis) [8–10]. A currently CMS approved test for informing lung cancer treatment is based on the former approach and specifically excludes the use of normal tissue (germline information) in determining somatic variants.

In contrasting the two approaches, this study analyzed tumor and normal DNA sequencing data from 45 lung cancer and 621 total cancer patients versus a tumor only gene panel approved for coverage by CMS. The study demonstrated a 94% false positive rate (95% for all cancers) when using tumor-only sequencing to identify somatic variants. Even after utilizing multiple methods for bioinformatically filtering polymorphisms from the putative somatic mutations, the false positive rates still ranged from 38%–95%. Depending on the method used, excessively stringent filtering led to potential false negatives. When focusing on a subset of 12 genes targeted by FDA-approved drugs, where identification of somatic mutations could inform treatment decisions, the percentage of lung cancer patients affected by false positive calls ranged from 29%–51% depending on the method of polymorphism filtration used. Further risk of false positive results stem from the identification of variants identified from somatic tissue, i.e., true somatic mutations misidentified as deleterious (inherited) germline variants in such genes as *BRCA1*, *BRCA2*, and *ATM*. In 10 genes associated with germline risk for familial disease (the inherited risk gene panel), true somatic mutations in germline genes were discovered in 10 lung cancer patients (11 variants) and 101 total patients (118 variants) when using the tumor-only sequencing approach. It may be concluded that sequencing and analysis of data from the patient’s normal germline genome and tumor genome eliminates false positive results associated with analysis of tumor genome sequence data alone.

Tumor-only sequencing will result in the identification of all variation present in the genome within the sequenced region, including germline and somatic variants. The results show that unambiguous identification of the somatic variants is not possible without the normal germline DNA sequence. The problem of variant origin identification is also present when tumor-only sequencing is used for germline variant calling; however, germline variant reporting is usually restricted to pathogenic and likely pathogenic variants found in ClinVar or an equivalent database and a misidentification of a somatic variant as a germline variant will most likely occur among variants of unknown significance. Focusing on the ten genes in the Inherited Risk Gene Panel, 3% of all variants found among lung cancer and all cancer patients were somatic mutations (Table 1). Without normal germline sequencing data, the 3% of variants that are somatic must be filtered from the germline variants if the goal is to identify inherited disease risk variants. Removing all variants that are not reported in dbSNP results in less than 1% of remaining variants within genes in the Inherited Risk Gene Panel being somatic for all cancer patients and lung cancer patients specifically.

To our knowledge, this is the first published study that assessed the increased precision achieved by adding RNA sequencing to a tumor-normal sequencing approach. The potential for tumor somatic SNVs to fruitfully inform patient treatment depends on expression of the DNA variants as messenger RNA, and then translation into protein. RNA sequencing of the tumor provides valuable information about relative expression levels of cancer driver genes, and the gene expression of specific tumor somatic variants [11–13]. RNA expression analysis in this study showed that 18% of true somatic mutations identified from tumor-normal sequencing of lung cancer patients, as well as 15% for all cancer patients, were not expressed at the level of messenger RNA. Analysis of true germline variants found that 9% and 11% of variants were not expressed among all cancer cases and lung cancer cases, respectively. The higher rate of unexpressed variants discovered in somatic versus germline is expected. Somatic mutations are novel to the cell and are more likely to result in transcriptional disruption or editing than germline mutations that have persisted through the life of the individual. Our results provide further evidence of the advantages associated with heightened precision of molecular analysis for drug targeting derived from tumor-normal DNA sequencing plus RNA sequencing.

The authors recognize that this study is limited by the number of lung cancer patients contributing variant data. Given that the tumors sequenced for this study were collected based on physician referral, they might not represent a true random sampling of the lung cancer population. Furthermore, clinical and demographic information to assess population representation was not available.

Simultaneous sequencing and bioinformatics analysis of the DNA of both the normal germline genome and the tumor genome is necessary for accurate identification of molecular targets for cancer therapy. Analysis of only the tumor genome results in a high false positive rate in SNV identification. Even higher precision is achieved with simultaneous tumor-normal DNA and RNA sequencing analysis. Treatment decisions based on tumor-only DNA analysis or in the absence of RNA analysis might result in administration of ineffective therapies while also increasing risk of negative drug-related side effects. When used to guide clinical decision-making, the approach of tumor-only gene-panel analysis may increase risk to patients, cause potential long-term negative health consequences, and increase healthcare costs.

## MATERIALS AND METHODS

### Patient samples

The patients that contributed samples that were used to generate the data used in this study had all provided informed consent to the use of their molecular information for research purposes. The patients had undergone genomic sequencing (whole genome tumor and normal DNA and tumor RNA) as part of an IRB approved research study or as prescribed by an authorized physician. The only selection criteria used for inclusion in the study was the completeness and quality of the genomic data and there were no exclusions based on tumor type, clinical or demographic factors. The study included 45 lung cancer patients and 621 total cancer patients with 30 different cancer types. The data are stored in the NantOmics, LLC database in a HITRUST certified environment.

### Sequencing data

Whole genome sequencing data from tumor DNA, tumor RNA, and normal DNA of 621 cancer patients was analyzed to identify somatically-derived single nucleotide variants (SNVs) potentially contributing to cancer growth and expansion. Although all patients samples had undergone whole genome tumor and normal sequencing, this study focused on mutation analysis in 35 genes. The panel included 25 genes implicated as somatic tumor drivers (tumor driver gene panel) and 10 genes that are known to affect inherited cancer risk (inherited risk gene panel). The tumor driver gene panel consists of: ALK, BRAF, CDKN2A, CEBPA, DNMT3A, EGFR, ERBB2, EZH2, FLT3, IDH1, IDH2, JAK2, KIT, KMT2A, KRAS, MET, NOTCH1, NPM1, NRAS, PDGFRA, PDGFRB, PGR, PIK3CA, PTEN, RET. The inherited cancer risk panel consisted of: APC, BMPR1A, EPCAM, MLH1, MSH2, MSH6, PMS2, POLD1, POLE, STK11.

DNA and RNA was extracted from preserved tissue and sequenced using the Illumina platform in a NantOmics Clinical Laboratory Improvement Amendments (CLIA)- and Certified Authorization Profession (CAP)-certified sequencing laboratory. Performance characteristics of the test used include > 95% sensitivity and > 99% specificity to detect SNVs transcribed and expressed as RNA. Normal germline and tumor genomes were sequenced to read depths of approximately 30× and 60×, respectively. Approximately 300 million RNA sequencing reads were generated for each tumor.

### Sequence alignment and variant calling

DNA sequencing data was aligned to GRCh37 (www.ncbi.nlm.nih.gov/assembly/2758/) by BWA [14], duplicate-marked by samblaster [15], and indel realignment and base quality recalibration performed by GATK v2.3 [16]. RNA sequencing data is aligned by bowtie [17] and RNA transcript expression estimated by RSEM [18]. Tumor vs. matched-normal variant analysis was performed using the NantOmics Contraster analysis pipeline to determine somatic and germline SNVs, insertions and deletions, and identify highly amplified regions of the tumor genome [8–10].

Small variants were annotated with base-level PhastCons conservation scores, population allele frequencies from dbSNP (Build 142), and their predicted impact to gene transcripts downloaded from the RefSeq database (eg, changes in DNA sequence and protein).

### Data analysis

Variants counts from DNA and RNA were calculated from VCF files generated by the bioinformatics pipeline. The true germline and somatic status variable was determined from NantOmics Contraster analysis of tumor and normal DNA sequence data and subsequently used to stratify variant counts. Variables used for variant filtering were downloaded from the dbSNP public database (https://www.ncbi.nlm.nih.gov/projects/SNP/). The calculations and data analysis presented in the Results section were carried out using Python, the R statistical package and C shell commands.
